# Supplementary material for: Social and environmental determinants of Neglected infectious diseases in quilombola communities of the Brazilian Amazon: An epidemiological and machine learning analysis
Source: PLoS Negl Trop Dis. 2026 Mar 4;20(3):e0014078. doi: 10.1371/journal.pntd.0014078 (PMC12970970; doi:10.1371/journal.pntd.0014078)
Supplement: Attachment — Submitted filename: Response to Reviewers.pdf [file pntd.0014078.s002.pdf]

Response to Reviewers

We thank the Editor and the Reviewers for their careful evaluation of our manuscript and for their constructive comments. We addressed each point raised and revised the manuscript accordingly. Below, we respond point by point and indicate the exact changes made, including the text added or modified.

Reviewer 1

Comment: The study objectives are clearly stated, and the hypothesis is well aligned with the research aims. The analytical cross-sectional design is appropriate. The population is well described, the sample size is adequate, the statistical methods are appropriate, and ethical approval is clearly reported. Only minor clarification regarding sample size calculation may strengthen the Methods section.

Response: We thank the reviewer for the positive assessment. To further strengthen methodological transparency, we added a brief description of the sample size calculation to the Methods section.

Text added to the Methods section: "The sample size was determined based on a sample size calculation using data from the total population of quilombola individuals in the municipality (N = 4,363)… This procedure indicated a minimum required sample of approximately 326 participants."

No other changes were required, as the reviewer confirmed that the manuscript meets the criteria for acceptance.

Reviewer 2

1. Lack of explicit testable hypothesis

Comment: The objectives are clear, but there is no explicit testable hypothesis.

Response: We agree and added a clear, testable hypothesis at the end of the Introduction.

Text added to the Introduction: "We hypothesized that the occurrence of NTDs in these communities is significantly associated with adverse socioenvironmental conditions, particularly inadequate sanitation, unsafe water sources, precarious housing, and low income, and that the combined analysis of these determinants through machine learning approaches would allow the identification of distinct vulnerability profiles and improve the prediction of disease risk."

2. Self-reported lifetime NTDs and recall bias

Comment: Because NTDs were measured through self-reported lifetime history, recall bias should be clarified and acknowledged.

Response: We clarified how NTDs were assessed and explicitly acknowledged recall bias in the Methods and Discussion.

Text added to the Methods section: "The occurrence of neglected tropical diseases was assessed based on self-reported lifetime history… Although this approach allows the capture of cumulative exposure, it may introduce recall bias, as past infections may not fully reflect current living conditions."

Text added to the Discussion (limitations paragraph): "The occurrence of neglected tropical diseases was assessed based on self-reported lifetime history, which may be subject to recall bias and may not fully reflect current exposure conditions."

3. Recruitment process and refusals

Comment: The recruitment process and number of refusals were unclear.

Response: We added a clear description of recruitment procedures and the number of refusals.

Text added to the Methods section: "Participants were recruited through active outreach in the selected quilombola communities… Of those invited, eight individuals declined participation."

4. Confounding, clustering, and inconsistencies in Table 6

Comment: Models were not adjusted for age, data were clustered by community, and crude ORs in Table 6 did not match raw counts.

Response: All main analyses were re-run adjusting for age and sex, with cluster-robust standard errors at the community level. Table 6 was fully recalculated to ensure consistency with observed frequencies.

Text added to the Methods section: "All models were adjusted a priori for age and sex… robust standard errors clustered at the community level were applied to account for intra-cluster correlation."

Correction implemented: Table 6 crude odds ratios now correspond exactly to the n/N proportions shown. Reference categories are explicitly labeled.

5. Machine learning methods insufficiently described

Comment: Variables, cross-validation, and class imbalance handling were under-explained.

Response: We expanded the Methods section to clearly describe the machine learning procedures.

Text added to the Methods section: "The study also included a cluster analysis using unsupervised machine learning techniques… Model training included data standardization, handling of class imbalance through class weighting, and stratified 5-fold cross-validation to ensure robust performance estimates."

6. Ethics statements, dates, and data availability

Comment: Informed consent statements were inconsistent, data collection dates conflicted, and the Data Availability Statement did not comply with PLOS policy.

Response: All ethical statements were harmonized, dates corrected, and the Data Availability Statement revised.

Corrected Informed Consent Statement: “Written informed consent was obtained from all participants prior to data collection.”

Corrected data collection period: “Data collection began on April 10, 2024, and continued through May 2025.”

Revised Data Availability Statement: “The data analyzed in this study are not publicly available due to privacy and ethical restrictions… researchers interested in accessing the data can contact the ethics committee.”

Reviewer 3

Definition of NTDs and “Any NTD” category

Comment: Malaria, hepatitis, and non-specific parasitic diseases should not be considered NTDs, and the “Any NTD” category requires clarification.

Response: We clarified the conceptual framework and analytical strategy in the Methods and Discussion.

Text added to the Methods section: “In addition to WHO-listed NTDs, diseases such as malaria, viral hepatitis, and parasitic infections were included due to their endemicity in the Amazon region… analyses were conducted at two levels: (i) Any NTD, capturing cumulative vulnerability, and (ii) disease-specific analyses.”

Text added to the first paragraph of the Discussion: “For transparency, WHO-listed NTDs are presented alongside regionally endemic conditions traditionally associated with neglect in the Amazon.”

Text added to the Results section: “Although the category ‘Any NTD’ aggregates diseases with distinct transmission mechanisms, it was used as a synthetic indicator of cumulative exposure… Disease-specific analyses were therefore presented.”

Results Section – Alignment and Presentation

Reviewer 2 concerns: Misalignment between Methods and Results, unclear reference categories, and figure quality.

Response: Methods were reorganized to align with Results.

All tables now explicitly label reference categories.

The spatial risk map was updated with higher resolution.

Conclusions and Limitations

Overstated causal language and missing analytical limitations

Comment: Causal claims were overstated, and key analytical limitations were not clearly acknowledged.

Response: We softened causal language and added a dedicated paragraph on analytical limitations.

Text added to the Discussion/Conclusion: The cross-sectional design of the study precludes causal inference… residual confounding cannot be ruled out… the relatively small number of events for some specific diseases may have reduced statistical power.”

Final Statement

We believe that all reviewer comments have been fully addressed with transparent revisions.
