# Supplementary material for: Social and environmental determinants of Neglected infectious diseases in quilombola communities of the Brazilian Amazon: An epidemiological and machine learning analysis
Source: PLoS Negl Trop Dis. 2026 Mar 4;20(3):e0014078. doi: 10.1371/journal.pntd.0014078 (PMC12970970; doi:10.1371/journal.pntd.0014078)
Supplement: Attachment — Submitted filename: Response Reviewers.pdf [file pntd.0014078.s003.pdf]

Dear Editor and Reviewers,

We would like to thank the Editor and all reviewers for their careful evaluation of our manuscript and for the constructive and insightful comments provided. We are grateful for the positive assessment of the study and for the opportunity to further improve the clarity, transparency, and consistency of the manuscript. Below, we provide a point-by-point response to each comment and detail the corresponding revisions made in the manuscript. All changes have been incorporated into the revised version.

Reviewer 1

Comment: The Method section is adequately described.

Response: We thank the reviewer for this positive evaluation. No additional changes were required in response to this comment.

Reviewer 2

Methods Comment: The study objectives are now clearly articulated and the addition of an explicit, testable hypothesis is a welcome improvement. The cross-sectional design is appropriate, and the population description is clear. The sample size is sufficient. However, clarification is needed regarding which population size was used in the sample size calculation and whether clustering by community was considered. A brief justification for the use of Poisson regression is also recommended. Additionally, the end date of data collection should be consistent across sections.

Response: We appreciate the reviewer's detailed and constructive feedback. The following revisions were made:

1. Sample size calculation: We clarified that the sample size calculation was based on the total quilombola population of the municipality and explained the rationale for this choice, given the territorial scope of the study. This clarification was added to the Methods – Sample size subsection.

2. Community clustering: We explicitly stated that although clustering by community was not incorporated at the planning stage of the sample size calculation, it was accounted for during the analytical phase through the use of robust standard errors clustered at the community level. This clarification has been added immediately following the sample size calculation.

3. Poisson regression justification: A brief methodological justification was added explaining that Poisson regression was used because the outcome represents a count variable (number of diseases per individual), allowing estimation of rate ratios for cumulative disease burden.

4. Data collection dates: We reviewed and standardized the data collection period across the Methods and Ethics sections to ensure full consistency and avoid potential confusion.

Comment: The Results section is improved and logically structured. However, the bivariate analysis table uses different categorizations of income and housing compared with multivariable models. Clarification or alignment is needed. A brief explanation distinguishing the aggregated outcome from disease-specific outcomes in the machine learning analyses is also recommended.

Response: We thank the reviewer for these helpful observations. Revisions were made as follows:

1. Bivariate vs. multivariable categorization: We added an explicit explanation in the Results section (immediately before Table 6) stating that bivariate analyses used more disaggregated categories for exploratory purposes, whereas aggregated categories were used in multivariable models to ensure statistical stability.

2. Aggregated outcome ("Any NID"): We added a clarification in the Results section introducing the machine learning analyses, explaining that the aggregated outcome represents a synthetic indicator of cumulative vulnerability and does not replace disease-specific analyses, which are presented in a complementary manner.

Additionally, minor refinements were made to figure labels, legends, and source attribution to improve clarity and visual consistency, particularly in the spatial risk map.

Conclusions

Comment: The conclusions are cautious and supported by the data. Public health relevance is clear. A concrete example of how findings may inform surveillance, resource allocation, or program prioritization would strengthen the section.

Response: We agree and have strengthened the Conclusions by adding a concrete example illustrating how the findings can support territorial prioritization of surveillance actions, targeted allocation of sanitation resources, and strengthening of primary care strategies in high-risk quilombola communities.

Comment: Include study limitations in the Author Summary. Improve consistency in terminology and refine the Data Availability Statement.

Response: Study limitations were explicitly added to the Author Summary, as requested. Terminology was carefully reviewed to ensure consistency throughout the text and tables, particularly regarding income, housing categories, and the disease terminology used. The Data Availability Statement was refined to clarify that anonymized data and metadata may be made available under controlled access upon reasonable request and ethics approval, in line with journal expectations.

Reviewer 3

Comment 1: The definition of NTDs differs from the WHO definition. A different term should be proposed, as only 16% of diseases align with the WHO NTD list.

Response: We thank the reviewer for this important observation. To avoid conceptual ambiguity, we replaced the term Neglected Tropical Diseases (NTDs) with Neglected Infectious Diseases (NIDs) throughout the manuscript. We added explicit definitions in the Introduction, Methods, and Discussion clarifying that this broader category includes both WHO-listed NTDs and other endemic infectious diseases that share similar social and environmental determinants of neglect in the Amazonian context. This change ensures conceptual clarity and directly addresses the reviewer's concern.

Comment 2: The exclusion criterion "visitors, temporary residents" requires clarification regarding minimum length of residence.

Response: We clarified the exclusion criteria by specifying that individuals with less than six months of continuous residence in the community were considered temporary residents and excluded from the study. This clarification has been added to the Methods.

Comment: Occupation appears as an important determinant in later analyses but is not included in the sociodemographic table.

Response: We clarified in the Results section that occupation was not included in the initial sociodemographic characterization due to its high heterogeneity and informality, but was incorporated into predictive models because of its discriminatory relevance in the context of territorial vulnerability. This explanation was added when presenting the variable importance results.

We believe that the revisions made fully address all reviewer comments and further strengthen the manuscript's clarity, methodological transparency, and conceptual consistency. We sincerely thank the Editor and reviewers for their valuable contributions and hope that the revised manuscript is now suitable for publication.

Sincerely,
The Authors
